# The Methods of Bacteriological Investigation

**Published:** 1886-08

**Authors:** 


THE METHODS OF BACTERIOLOGICAL INVESTIGATION. By Dr. Ferdinand Hueppe, Docent in Hygiene and Bacteriology. Translated by H. M. Biggs, M. D., Instructor in the Carnegie Laboratory, and Assistant to the Chair of Pathological Anatomy in Bellevue Hospital Medical College. New York: D. Appleton & Co. 1886.

Of the many works that have recently appeared on the subject of bacterial technology, this one certainly meets the requirements of a practical guide and book of reference. The best methods are presented in a clear, concise and practical manner, and with such details as to render them comprehensible. The translation is generally good, but in some places too close adherence to German idiom somewhat obscures the sense. But the merits of the work are decided, and should secure for it the reputation it deserves.

